# Effects of urban sprawl on arthropod communities in peri-urban farmed landscape in Shenbei New District, Shenyang, Liaoning Province, China

**DOI:** 10.1038/s41598-017-18474-9

**Published:** 2018-01-08

**Authors:** Zhen-xing Bian, Shuai Wang, Qiu-bing Wang, Miao Yu, Feng-kui Qian

**Affiliations:** 1 College of Land and Environment, Shenyang Agricultural University, Shenyang, 110161 China; 2 College of Science, Shenyang Agricultural University, Shenyang, 110161 China

## Abstract

Peri-urban farmland provides a diversity of ecological services. However, it is experiencing increasing pressures from urban sprawl. While the effects of land use associated with farming on arthropod assemblages has received increasing attention, most of this research has been conducted by comparing conventional and organic cropping systems. The present study identifies the effects of urban sprawl and the role of non-cropped habitat in defining arthropod diversity in peri-urban farmed landscapes. Multi-scale arthropod data from 30 sampling plots were used with linear-mixed models to elucidate the effects of distance from urban areas (0–13 km; 13–25 km and >25 km, zones I, II, and III, respectively) on arthropods. Results showed that urban sprawl, disturbed farm landscapes, and disturbance in non-cropped habitats had negative effects on arthropods, the latter requiring arthropods to re-establish annually from surrounding landscapes via dispersal. While arthropod species richness showed no obvious changes, arthropod abundance was lowest in zone II. Generally, patch density (PD), Shannon diversity index (SHDI), and aggregate index (AI) of non-cropped habitat were major drivers of changes in arthropod populations. This study contributes to identifying the effects of urban sprawl on arthropod diversity and documenting the multiple functions of farm landscapes in peri-urban regions.

## Introduction

Asia has become strongly urbanized with 60% of the world’s megacities, those defined as cities having a metropolitan area with more than 10 million inhabitants, located in Asia^1^. These cities create strong and ever-increasing pressure on farmland. The urban population of Asia is expanding by more than 45 million residents annually, and this causes a loss of agriculturally productive land of more than 10 km^2^ d^−2^. Growing metropolitan regions often penetrate important agricultural areas in emerging peri-urban zones where rural and urban human activities are closely intermingled to form a seemingly chaotic land use pattern^2,3^ and create an increase need for food as well as causing poverty and environmental degradation^4,5^. Recent studies have shown that the spatial configuration of farmland in peri-urban areas can improve environmental quality^6^ and help to create an amenity-rich, biodiverse landscape, and to provide outdoor spaces for the urban population^7,8^. However, the decline of biodiversity in this type of peri-urban farmland has altered the provisioning of ecological services in agricultural ecosystems; these ecosystems mediate energy and material fluxes or alter abiotic conditions^9^. But these ecological services suffer in the considerably simplified landscape pattern caused by urban sprawl and the progressive intensification of agriculture^10^.

Related research has shown that farm landscapes surrounding cities tend to become fragmented and discrete units that suffer from homogenization^11^ with decreasing non-cropped habitats^12^. These non-cropped habitats are primarily comprised of trees, hedgerows, and grassy margins and their amount, quality, and spatial configuration can have strong implications for the delivery and sustainability of various ecosystem services^13–15^. The presence of a complex landscape with a high proportion of non-cropped habitat is more beneficial than uniform landscapes for maintaining biodiversity, and the focus of attention and research is currently on the effects on biodiversity from the proportion^16^ and diversification^17^ of non-cropped habitats. Conditions in non-cropped habitats may provide a wider variety of resources for arthropods than the crop fields^18^. Retaining viable arthropod populations in farmed landscapes in critical for sustaining the provision of ecosystem services^19^. Non-cropped habitats in peri-urban farmed mosaics may help to maintain landscape heterogeneity and sustain diverse arthropod assemblages^20^. Characterizing the dynamic changes of urban sprawl that has affected on landscape patterns of non-cropped habitat in turn has affected arthropods assemblage in peri-urban farmland is important. However, rather few studies have showed the feature of peri-urban farmland arthropod biodiversity in process of urbanization^21^. In addition, relationship of arthropod biodiversity and the pattern of non-cropped habitat which effected by urban sprawl remain poorly understood^22^.

The main objective of our research was to assess the value of non-cropped habitats that were affected by urban sprawl for sustaining arthropod diversity in peri-urban farmed mosaics. In addition, our study was also aimed at identifying the landscape metrics associated with the spatial heterogeneity of farmland at different distances from a city centre, a factor that influences the structure of arthropod assemblages. The presence or absence of non-cropped habitat in an intensively farmland landscape significantly affects the biodiversity of arthropods^23^. Thus, arthropod assemblages may vary in different farmland landscapes that are affected by urban sprawl. It could be hypothesized that arthropod assemblages should be the lowest in most urbanized areas. Based on this hypothesis we analyse: (1) the effects of the intensity of urbanization at multiple scales (i.e. inter-urban fringe, outer-urban fringe, rural areas) on the species richness and abundance of arthropods, (2) the importance of non-cropped habitats to retaining arthropod diversity, and (3) the relationship of farm landscape metrics and arthropod diversity. We expect the findings of this paper to help improve the development of specific approaches to the controlled development of mixed urban-rural landscapes to support the sustainability of urban areas.

## Methods

### Study site

The study, conducted in peri-urban regions of Shenbei New District (41°54′–42°11′N, 123°16′–48′E) in Shenyang city, Liaoning, China, covered a total area of 10,980 km^2^ and has an average elevation of 58 m. The continental monsoon climate in this north temperate zone features four distinct seasons with a mean annual temperature of 7.5 °C and mean annual precipitation of 672.9 mm. The peri-urban regions of Shenyang have continued to extend outward and have been powerfully disturbed by cities and humans with an acceleration of urbanization since the establishment of Shenbei New District in 2006. Shenbei New District is a typical pri-urban area in northeast, China.

**Figure 1:**
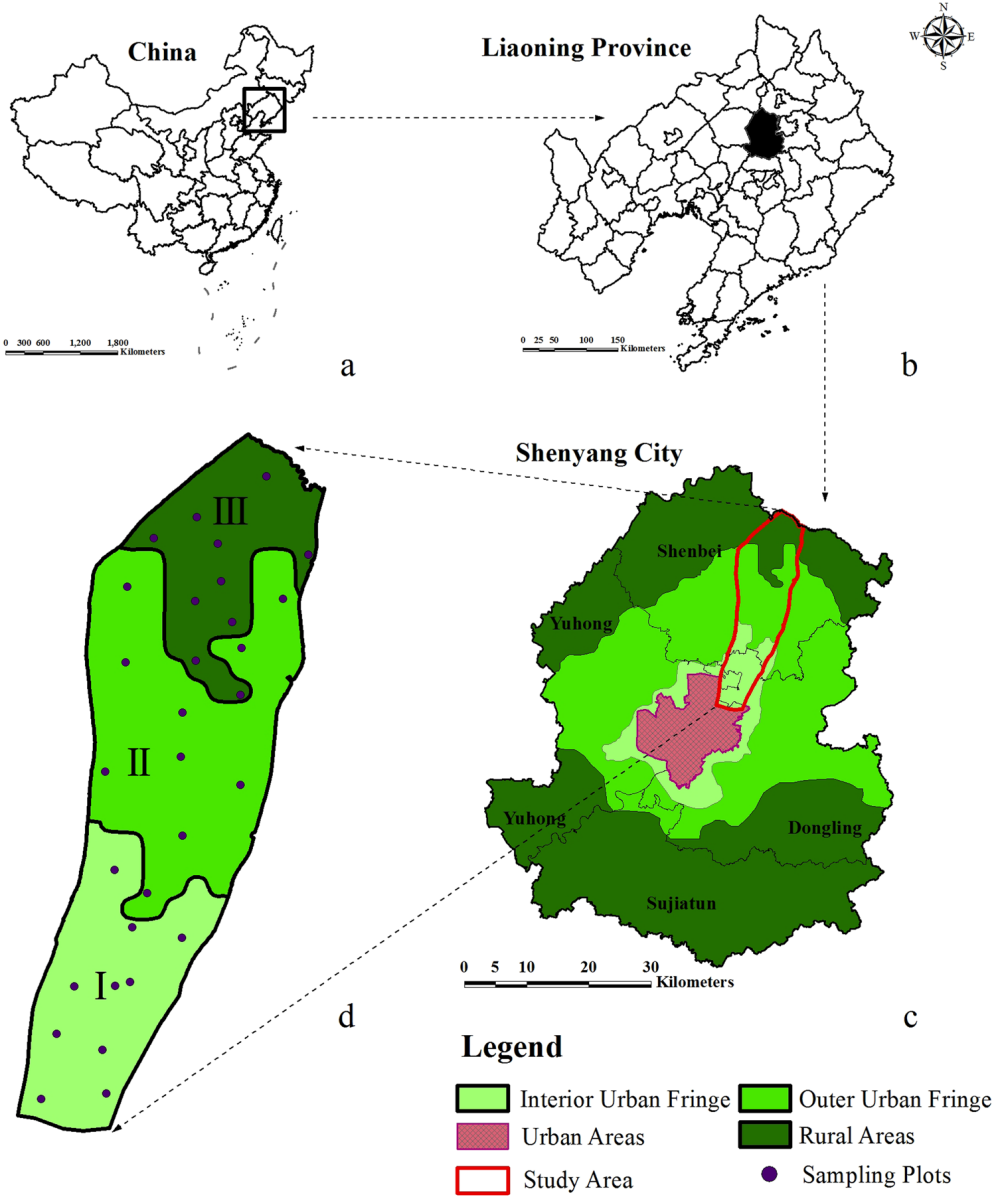
Study areas and location of the sampling plots. (**a**) vicinity map showing the location of Liaoning Province China; (**b**) vicinity map showing the vicinity of Shenyang within Liaoning; (**c**) vicinity map showing the location of Shenyang, local administrative areas, and the study area; (**d**) map of study sites within the study area. The figure was generated by using ArcMap 10.0 (http://www.esri.com/).

For the present study, Shenbei New District was divided into three regions based on landscape disturbance density models^24^ (Fig. 1) from Chen & Zhao 1995^25^. Those regions were called the inter-urban fringe, outer-urban fringe and rural areas, respectively based on entropy values of land use intensity. The regions with entropy values greater than 0.8 were called the outer-urban fringe; the leading edges of urban expansion were the most intensely affected areas and were near urban areas with the dual characteristics of urban and rural landscapes. The regions with entropy less than 0.8 were separately classified as inter-urban fringe with dominant urban and a few rural characteristics. Rural areas were far from the city) and had little or no influence from urban areas based on their land-use types.

### Sampling of arthropods

Surveys were performed during the summer (13–28, June) and autumn (15–30, Sep.) of crop growing season in 2013 and 2014, in a typical peri-urban region, selected from urban areas of Shenyang city toward to the administrative boundary of the Shenbei New District. These survey sites were parts of three zones: the inter-urban fringe (herein called zone I and covering 5311 ha), outer-urban fringe (herein called zone II, covering 12,692 ha), and rural areas (herein called zone III, covering 5335 ha), within 0–13 km, 13–25 km, and >25 km from urban areas of Shenyang, respectively. The main non-cropped habitat types were classified as either grassland (headland, road, ridge, and drainage ditch) or woodland (fencerows, tree belt, and woodlot) based on vegetation form.

Arthropods were surveyed in 30 sampling areas (ten areas in each zone) that were selected randomly. Each area had three sampling points, one each in arable land, grassland, and woodland. Three independent 25 × 25 cm quadrants were selected as repetitions for each sampling point^24^.

As much as possible, potholes, earth mounds, slope, rocksloping areas, rocks, roots, and so on were avoided. The method of Hand picking was adopted to takefor collecting samples of arthropods from three land-cover classes: arable land, grassland, and woodland. The sample specimens were placed into a 75% ethanol collection bottle brought backbottles and returned to the laboratory for identification^26^. Arthropods were taxonomically identified at the order level in all cases and at the lowest taxonomic level when possible (i.e., family, genus, or species). Species richness of arthropods was expressed as the mean number of taxa at each sampling point. Arthropod abundance was also measured.

### Farm landscape heterogeneity

Landscape heterogeneity was described by calculating the landscape metrics of land-cover types surrounding each field with sampling points. Landscape metrics included patch density (PD, number of patches per unit area, e.g., per km^2^), large patch index (LPI, ratio of the area of the largest patch to the total area of the landscape, unit: %), edge density (ED, total length of all edge segments per ha for the class of landscape of consideration, unit: m/ha), landscape shape index (LSI, a modified perimeter area ratio of the total length of patch edges and the total area of the landscape), Shannon diversity index (SHDI, patch diversity in a landscape that is determined by both the number of different patch types and the proportional distribution of area among patch types)^27^ and, aggregate index (AI, ratio of the observed number of like adjacencies, based on the single-count method, to the maximum possible number of like adjacencies given the proportion of the landscape comprised of the focal class)^28^. Three land-cover classes were identified including arable land, grassland, and woodland. Classification was based on the Current Land Use Classification in China (GB/T21010-2007). Land use data (1:10000) for 2012 was obtained from the Shenbei Land Resource Bureau.

The landscape metrics listed above were computed using a FRAGSTATS^29^ (http://www.umass.edu/ landeco/research/fragstats/documents/fragstats.help.4.2.pdf) interface with QGIS (www.qgis.org) software. Both landscape- and class-level metrics were computed using land cover grids based on the eight-cell rule for patch neighbours, which considers both orthogonal and diagonal cells as neighbors^30^.

### Statistical analysis

Differences in arthropod richness and abundance in the different regions were compared by using a sample-based rarefaction procedure, where individuals are set as samples and curves are then calculated by using the Mao Tau estimator^31^. The significance of observed differences in arthropod richness and abundance between the three urbanization regions analysed in the present study (*P* < 0.05) was evaluated by visually comparing rarefaction curves and their associated 95% confidence intervals. Estimated arthropod richness and abundance were calculated by using an abundance-based coverage estimator^32^.

The explanatory factors that have contributed to arthropod diversity and abundance were analysed with linear mixed-effects models^33^. Mixed models were used to test for the effects of urbanization within the three zones (I, II and III). Sampling times were not used in the models as co-variables without having significant effects in the arthropod richness and abundance. Sampling points within each region were included as random variables in the mixed-effected models to account for the experiment design. Landscape metrics were included in the model as fixed explanatory variables at each zone. Fixed effect variables were the habitat types within fields, including arable land, grassland and woodland. A simplification model was applied by first removing the non-significant interaction terms (F test, *P* > 0.05). Subsequently, non-significant main effects were removed only when they were not involved in a significant interaction^34^. Spatial autocorrelation analysis showed no significant values for Moran’s I Coefficient for richness and abundance between plots (I = −0.0985, *P* = 0.2022), because linear mixed effects models were applied to each plot individually. All analyses were performed with R software.

## Results

### Changes in landscape patterns of non-cropped habitats

**Figure 2:**
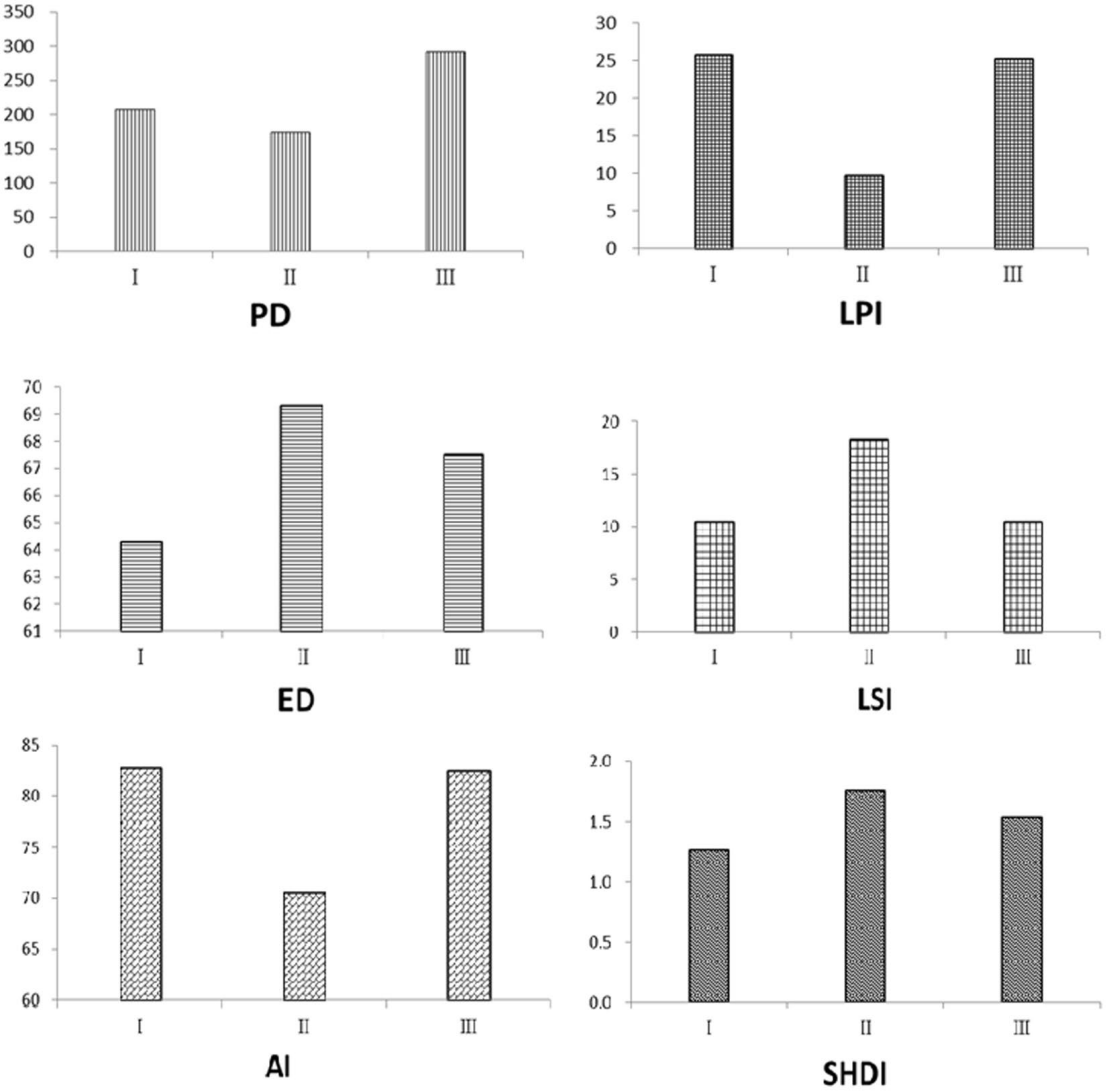
Non-cropped habitat landscape metrics; (a) patch density (PD); (b) large patch index (LPI); (c) edge density (ED); (d) landscape shape index (LSI); (e) aggregate index (AI); (f) Shannon diversity index (SHDI).

According to the Fig. 2, the PD, LPI, and AI initially decreased from zones I to III, and then increased. However, ED, LSI, and SHDI increased from zones I to III, and then decreased. When the six landscape matrices of the non-cropped habitats were compared, the results demonstrated that non-cropped habitats in zone II were more isolated, regular, dispersed, and diverse than those in zones I and III. Most new urbanization occurring in zone II was concentrated in farmland, because farmland was believed to be physical suitable for new urbanization. New urbanization gradually occupied farmland and thus fragmented the non-cropped habitats in zone II. However, the residual farm landscape in zone I or farmland that had been planned as a future urbanized landscape was stable. Meanwhile, urban sprawl had no or only a slight impact on the farm landscape in zone III. In zone II, disturbance caused by urbanization commonly impacted the non-cropped habitat landscape at the rural-urban interface. Thus, urbanization played an important role in shaping the landscape patterns of non-cropped habitat.

### Species of arthropods

A total of 18 species were identified from the 3102 arthropod specimens captured (Table 1). Arthropods consisted of the following five orders: Coleoptera, Collembola, Araneae, Geophilomorpha, and Opiliones, which contained 56.74%, 17.83%, 6.38%, 8.61%, and 10.44% of all arthropod specimens, respectively. The Coleoptera had fourteen families, including the Staphylinidae, Curculionidae, and Scaphidiidae among others. Additionally, 11 orders and families of arthropods were common including those of the Staphylinidae, Curculionidae, Scaphidiidae, Scydmaenidae, Histeridae, Silphidae, Pselaphidae, Collembola, Araneae, Geophilomorpha, and Opiliones. Three families of arthropods were rare including the Silvanidae, Cucujidae and Tetrigoidea. The dominant taxa included 3030 individuals and accounted for 97.68% of the total number of individuals in the study zones samples, which constituted the main component of arthropods and played an important role in ecosystem resource cycling^17^.

**Table 1:** The populations of four orders and 14 families of arthropods.

Orders	Families	Populations	(%) Individual to total	Abundance
Coleoptera	Staphylinidae	583	18.79	+++
	Curculionidae	316	10.19	+++
	Scaphidiidae	144	4.64	++
	Scydmaenidae	174	5.61	++
	Histeridae	114	3.68	++
	Silphidae	96	3.09	++
	Pselaphidae	78	2.51	++
	Erotylidae	54	1.74	++
	Carabidae	39	1.26	++
	Chrysomelidae	54	1.74	++
	Lathridiidae	36	1.16	++
	Silvanidae	24	0.77	+
	Cucujidae	24	0.77	+
	Tetrigoidea	24	0.77	+
Collembola		553	17.83	+++
Araneae		198	6.38	++
Geophilomorpha		267	8.61	++
Opiliones		324	10.44	+++
Total Numbers		3102	100	

^+++^Dominant population (>10%), ^++^Common population (1–10%), and ^+^Rare population (<1%).

### Accumulation curves for arthropod species and individuals

**Figure 3:**
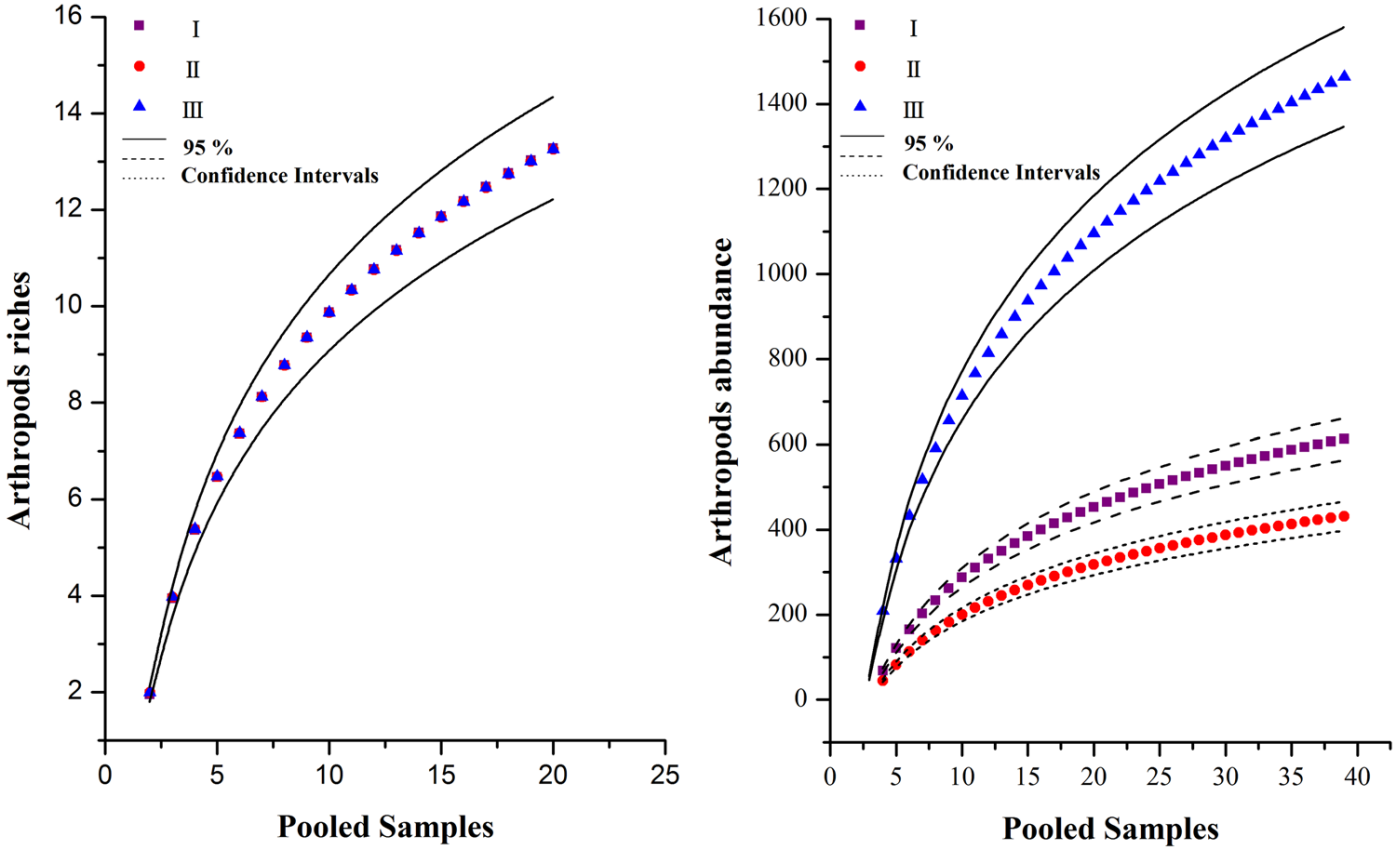
Accumulation curves of arthropod richness and abundance in different urbanization zones. Left: arthropods richness: I y = 4.5641*ln(x) − 0.9939, R² = 0.8066; II y = 4.8905*ln(x) − 1.3956, R² = 0.8432; III y = 4.9195*ln(x) − 1.4573, R² = 0.8729. Right: arthropod abundance: I y = 239.15*ln(x) − 263.51, R² = 0.7437; IIy = 169.95*ln(x) − 191, R² = 0.749; III y = 550.95*ln(x) − 555.11, R² = 0.8003).

The accumulated number of arthropod taxa was similar between the three zones. Only four, four, and six taxa were captured only once at each sampling position across all sample plots in zones I (31% of 13 sp.), II (29% of 14 sp.), and III (43% of 14 sp.), respectively. The accumulation curve of the three regions had similar increasing rate of species numbers found per zone (Fig. 3). Meanwhile, the species accumulation curves for the three zones had a close approximation increment of species number per zone.

Patterns of accumulated numbers of arthropod individual differed between the three regions. The percentage of arthropod individual in all sample were 25.53% in zone I, 17.99% in zone II, and 56.48% in zone III. The accumulation curve corresponding to zone III differed from those of zones I and II; these three zones also had different rates of increase in the numbers of arthropod individuals in each sampling plot (Fig. 3). However, the individual accumulation curve for zone III had a significantly higher increment for the number of individuals in each sampling plot than the other two zones.

### Arthropod species richness

**Figure 4:**
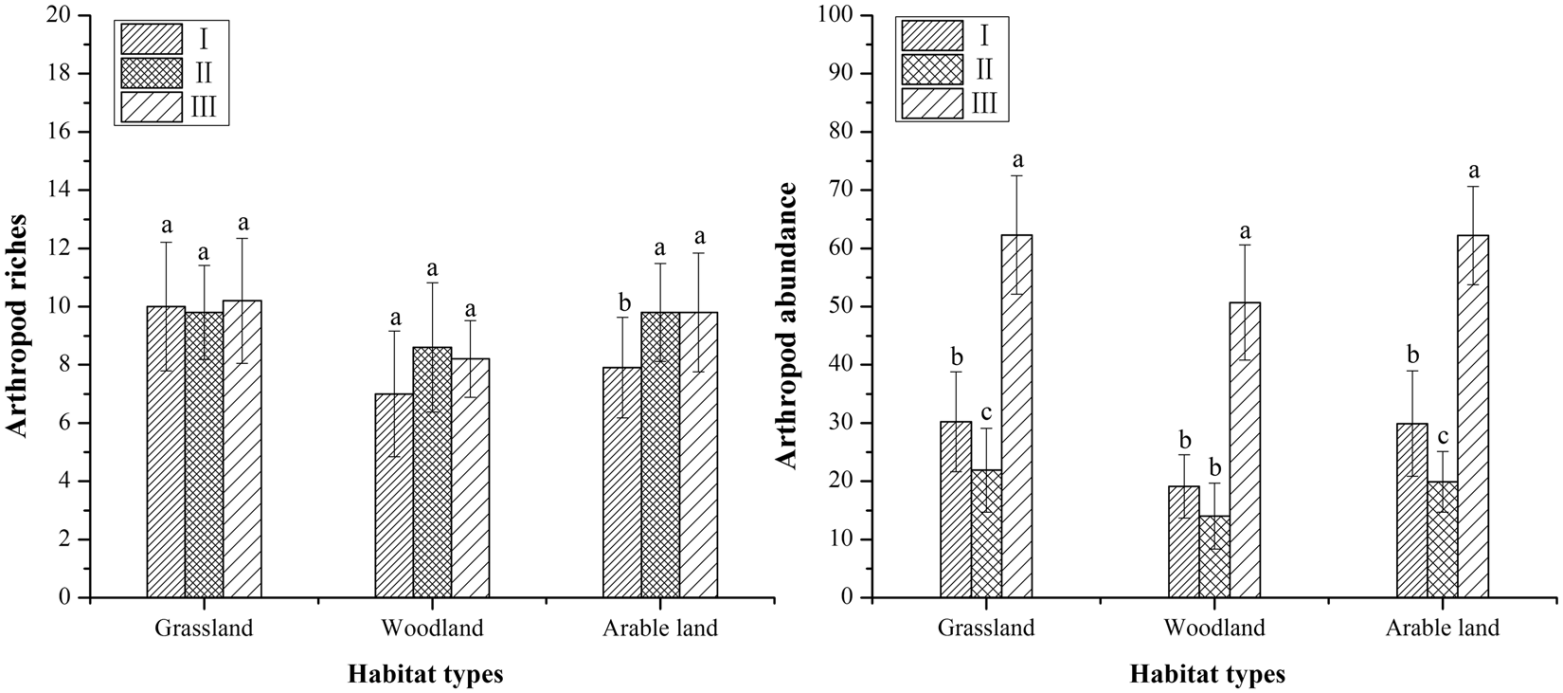
Mean (left panel) arthropod richness and (right) abundance (±Standard Error) in zones I (0–13 km from the city edge, II, (13–25 km), and III (>25 km) in grassland, woodland, and arable land.

The species richness of arthropod assemblages has no obvious change in three zones (Fig. 4a). Meanwhile, Arthropod richness was very similar in grassland (10 ± 2.2 sp., 9.8 ± 1.6 sp. 10.2 ± 2.1 sp.) in all three zones (Fig. 4a). There were significant correlations between arthropod species richness and PD, LPI, ED, LSI, SHDI, and AI of non-cropped habitats in zones I and III, whereas there were the same correlations in zone II except for LPI (Table 2). There were significant interactions between the factors included in the models (*P* <0.05). Regarding the landscape metrics of non-cropped habitats in zones II and III, different landscape patterns maintained similar levels of richness of arthropods. Generally speaking, arthropod richness in zone I was similar to that in zones II and III. Thus, arthropod species richness was negatively associated with urban sprawl in both heterogeneous and homogeneous non-cropped habitats.

**Table 2:** *F* values and levels of significance of linear mixed models testing the effects of non-cropped habitat metrics for arthropod richness and abundance.

Source	Arthropods richness			Arthropods abundance		
	d.f.	F	P value	d.f.	F	P value
I						
PD	29.056	80.483	<0.0001	29.863	65.11	<0.0001
LPI	32.041	91.324	<0.0001	58	1.791	0.901
ED	29.713	46.562	<0.0001	39.559	15.441	<0.0001
LSI	58	3.28	<0.0001	30.914	52.546	<0.0001
SHDI	29.8	45.513	<0.0001	29.052	75.253	<0.0001
AI	34.074	19.489	<0.0001	58	1.845	<0.0001
II						
PD	29.112	63.527	<0.0001	30.456	48.598	<0.0001
LPI	58	0.782	0.601	35.195	20.582	<0.0001
ED	29.395	120.177	<0.0001	34.083	65.314	<0.0001
LSI	43.543	39.485	<0.0001	44.381	6.34	0.83
SHDI	29.016	54.284	<0.0001	29.001	50.633	<0.0001
AI	33.452	112.745	<0.0001	58	1.319	<0.0001
III						
PD	29.022	255444.656	<0.0001	29.627	6151.803	<0.0001
LPI	46.25	7.261	<0.0001	29.672	37.405	<0.0001
ED	29.227	22963.642	<0.0001	35.388	382.135	0.149
LSI	58	2.741	0.013	29.937	35.32	<0.0001
SHDI	29.196	61.851	<0.0001	29.007	48.667	<0.0001
AI	46.551	77.627	<0.0001	34.987	17.652	<0.0001

Note: PD, patch density; LPI, large patch index; ED, edge density; LSI, landscape shape index; SHDI, Shannon diversity index, AI, aggregate index.

### Arthropod abundance

The patterns of arthropod abundance were obviously different to those observed for species richness. Zone III had the greatest abundance of arthropod individuals, while this was significantly lower in zone II and the increased slightly in zone I. In arable land, arthropod abundance was higher in zone III (62.2 ± 8.4) than in zones I (29.9 ± 9) and II (19.9 ± 5.2) (Fig. 4b). In grassland, arthropod abundance was highest in zone III (62.3 ± 10.2), followed by zones I (30.2 ± 8.6) and II (21.9 ± 7.2). As had been observed in both arable land and grassland, arthropod abundance in woodland was highest in zone III (50.7 ± 9.9) and lowest in zone II (14 ± 5.7) (Fig. 4b). Overall, in arable land, grassland, and woodland, arthropod abundance exhibited the same trend when moving from zone I toward zone III. Based on the landscape metrics of non-cropped habitats arthropod abundance remained higher in zones I and III than in zone II. However, the high heterogeneity of non-cropped habitats in zone II did not allow zone II to maintain the highest abundance of arthropods. There were significant correlations between arthropod abundance and PD, SHDI and AI in all three zones, whereas there was no significant correlations with LPI in zone I, LSI in zone II, or ED in zone III (Table 2). Thus, the landscape pattern of peri-urban farmland that had been disturbed by urban sprawl had no discernible effect on the abundance of arthropods.

## Discussion

Developing ecological corridors that are designed to connect residual natural and semi-natural habitats in the farm landscape could help to increase landscape connectivity and to conserve biodiversity in a larger range of farm landscapes^35^. The present study demonstrated that the isolation of non-cropped habitats contrasted with agricultural biodiversity conservation. In the present study zones, I, II, and III were experiencing different stages of urbanization. That is, the intensity of disturbance varied in different areas based on the nature of ongoing urbanization, which was affected by the city of Shenyang during its northward expansion. Farmland in zone I had already established a new micro-farm landscape pattern under the background of the landscape scale pattern of urbanization. Zone I had experienced strong levels of disturbance from urbanization and this disturbance gradually tended to create a new ecosystem, which gave the non-cropped habitat a certain level of connectivity and complexity. Zone I was in the formation stage for the corresponding relationship between the proportion of non-cropped habitat patches and individual number of arthropods in zone I. Most areas of zone III were still experiencing weak or none urbanization, where the farm landscape was barely affected by urban sprawl. In zone III the original and relatively stable farmland landscape was being maintained, and relatively strong connectivity and complexity remained in the non-cropped habitats of zone III. Therefore, the present study resulted in the same conclusion of another study that showed heterogeneity was associated with non-cropped habitats as a key driver of arthropod population in farmland mosaics^18^. Zone II was experiencing intense urbanization. Here the non-cropped habitats were unstable because urbanization constantly fragmented and disturbed farmland, which has resulted in disturbance of the landscape pattern of non-cropped habitats. Although zone II has relatively more non-cropped habitat patches in an unstable farm landscape, most were not suitable for arthropod survival, which has been a main cause of decreased arthropod abundance.

The landscape pattern of non-cropped habitats is shown in Fig. 2, while Table 2 demonstrates the relationships between the metrics of non-cropped habitats and arthropod abundance. PD, LPI, and AI were lower in zone II than in zones I and III. Meanwhile, ED, LSI, and SHDI were higher in zone II than in zones I and III. In six landscape metrics, LPI in zone I, LSI in zone II, and ED in zone III each independently had no significant correlation on arthropod abundance. These relationships demonstrate that PD, LPI and AI were important contributors to a decrease in arthropod abundance in zone II. Patch diversity, defined as ED, LSI, and SHDI, had less of an influence on arthropod abundance than did PD, LPI, and AI. The spatial configuration and arrangement of the landscape pattern of non-cropped habitats plays an important role in biodiversity and ecosystem functioning^36,37^. The disturbance, displacement, and remodelling of landscape structure in non-cropped habitats by urbanization to not all the high level of heterogeneity of farmland to be maintained, which affected the behaviour and movement of arthropods^38,39^. In zone I, the process of urbanization in the city had been completed, the remnant of farmland was small and relatively stable as a farm landscape embedded within the urban system, which allowed zone I to retain a relatively high level of patch density and connectivity. Retaining a landscape pattern with non-cropped habitats may help to maintain the heterogeneity of the farm landscape and, therefore may also help to sustain diverse arthropod assemblages. The abundance of arthropods decreased with the decrease in the diversity of non-cropped habitats in zone II because human activities had intensely disturbed the landscape structure of non-cropped habitats, and then reduced the function of non-cropped habitats as refuges and food resources. Furthermore, dispersion and fragmentation of non-cropped habitats resulted in a more intense decrease in arthropod abundance in zone II. In zone III, the farmland mosaics comprised of different non-cropped habitats usually supported relatively high levels of overall arthropod abundance, which resulted from the traditional agriculture structure that had experience no or little influence from urban sprawl. Therefore, in zone III future urban sprawl may disturb the farm landscape and result in a further decrease in arthropod abundance.

It is worth noting an obvious change in arthropod abundance had occurred, although there was no obvious change in arthropod species richness in zones I to III. The field investigation revealed that all three zones had the same non-cropped habitat types. The non-cropped habitat type was a major determinant of arthropod species richness in the peri-urban farm landscape because this type of habitat had similar micro-environmental conditions prevailing in all three zones.

## Conclusions

In our study, the non-cropped habitat landscape pattern in zone II was more isolated, regular, dispersed, and diverse than similar landscape patterns in zones I and III. Arthropod richness had no obvious change from zones I to III. Arthropod abundance was lowest in zone II, and was significantly lower than in zone III, in rural areas. Therefore, we found that urban-sprawl had negative effects on arthropods, particularly those associated with disturbed non-cropped habitats and weakened farmland heterogeneity. The disturbance dynamics in non-cropped habitats require arthropods to re-establish their populations in these areas annually from surrounding landscape via dispersal^17^. Accordingly, after a disturbance caused by changing stages of urbanization, arthropod species may persist in peri-urban farm landscape through meta-population effects. Nevertheless, we do not know which groups of arthropods species may suffer or benefit from the habitat alteration effected by urbanization. In this regard, future research that identifies species with different traits will be able to provide a finer understanding.

Human activities in peri-urban areas had a clear and significant impact on farmland ecosystems, and the speed of human interference was much larger than the speed of recovery in farm ecosystems with an increase in non-cropped habitats during urbanization. Hence, in order to maintain farm biodiversity and improve the ecological quality of cultivated land it is often necessary to increase the diversity and complexity of farm landscapes. This can be done by simultaneously restricting urban expansion and increasing the proportion of non-cropped habitat areas. To increase the availability of non-cropped habitat, it may be necessary to have clear limits on land use. For example, urbanization may need to be limited in each area to maintain a desired proportion and type of landscape structure in non-cropped habitats.

Additionally, a need may exist to monitor and evaluate the ecological quality of farmland and the multi-functional state of habitats that surround cities. This can be done by considering arthropods and other species groups as biodiversity indicator organisms during farmland urbanization, such as monitoring land consolidation, the development of primary cultivated land and so on, in the future.
